# An innovative state-of-the-art health storytelling technique for better management of type 2 diabetes

**DOI:** 10.3389/fpubh.2023.1215166

**Published:** 2023-09-29

**Authors:** Sumbal Ali, Shumaila Bakht, Atta Ullah Jan, Iftikhar Alam, Ali Madi Almajwal, Tareq Osaili, Reyad Shakir Obaid, MoezAllslam Ezzat Faris, Leila Cheikh Ismail, Farah Najah, Hadia Radwan, Haydar Hasan, Mona Hashim, Sharifa AlBlooshi, Bismillah Sehar, Falak Zeb

**Affiliations:** ^1^Human Nutrition and Dietetics, Bacha Khan University, Charsadda, KPK, Pakistan; ^2^Department of Agriculture (Human Nutrition and Dietetics), Bacha Khan University, Charsadda, Pakistan; ^3^Department of Community Health Sciences, College of Applied Medical Sciences, King Saud University, Riyadh, Saudi Arabia; ^4^Research Institute of Medical and Health Sciences, University of Sharjah, Sharjah, United Arab Emirates; ^5^Department of Clinical Nutrition and Dietetics, College of Health Sciences, University of Sharjah, Sharjah, United Arab Emirates; ^6^Department of Nutrition and Food Technology, Faculty of Agriculture, Jordan University of Science and Technology, Irbid, Jordan; ^7^Department of Women's and Reproductive Health, University of Oxford Nuffield, Oxford, United Kingdom; ^8^College of Natural and Health Sciences, Zayed University, Dubai, United Arab Emirates; ^9^Department of Health and Social Sciences, University of Bedfordshire, Luton, United Kingdom

**Keywords:** storytelling technique, type 2 diabetes, health education, innovative health techniques, health story

## Abstract

**Background:**

Type 2 diabetes (T2D) is a chronic lifelong disease that requires long-term prevention and management strategies in a community setting. A health story is a novel technique that may be used as an effective tool for better prevention and management of T2D.

**Objective:**

The main objective of this study is to develop a story to be used as a social health technique based on contemporary scientific knowledge that may be used at a community level for better communication and management of T2D.

**Methods:**

A community–academic partnership was formed with a not-for-profit Nutrition Education, Awareness, and Training (NEAT) organization in Khyber Pakhtunkhwa, Pakistan. We agreed to develop a story that may be used as a health and nutrition education communication tool for better management of patients with T2D. The following phases were followed during the story creation process: (1) the theory phase, (2) the modeling phase, and (3) the evaluation phase. Raters evaluated the story to determine its literary and scientific quality, comprehensiveness, and T2D specificity.

**Results:**

The title of the story translated into English is “The Story of Diabetes—The Story of Success.” It is text based and contains 86 pages in the local language, “Pashto,” with an English translation. The story is divided into five chapters and describes the initial diagnosis, fear associated with the disease, issues related to referral to certified practitioners, the importance of a balanced diet, and related lifestyle habits. After story evaluation, the raters suggested its literary and scientific quality, comprehensiveness, and T2D specificity (Pearson correlation scores of >0.8).

**Conclusion:**

This unique story was created for T2D and found to be of significant quality in terms of its literary and scientific quality, as well as its comprehensiveness and diabetes specificity. As a result, it may be suggested that it can be used in subsequent studies to improve T2D management among adult patients.

## 1. Introduction

Health education is a vital part of any healthcare policy process that combines learning experiences designed to help individuals and communities improve their general health. This is primarily done by increasing the knowledge and fostering positive attitudes of the community. The World Health Organization (1) emphasizes that the goal of health education is to foster the motivation, skills, and confidence (self-efficacy) necessary to improve health. Another goal is to communicate information concerning the underlying social, economic, and environmental conditions impacting health (1). A broad purpose of health education, therefore, is not only to increase knowledge about personal health behavior but also to develop skills that “demonstrate the political feasibility and organizational possibilities of various forms of action to address social, economic and environmental determinants ofhealth” (1, 2).

Health narratives and storytelling (ST) emerge as useful tools in health education and communication (3–7). Andreae et al. (5) developed and successfully implemented an intervention that emphasized peer modeling and support as strategies to improve medication adherence in diabetes patients. Zarifsaniey et al. (4) concluded that employing digital storytelling for diabetes self-management training can considerably enhance the self-management skills of adolescents with type 1 diabetes. Lohr et al. (3) concluded from their findings that the storytelling intervention protocol provides a template for future health promotion interventions that prioritize health disparity population behaviors. Leveraging the insights shared by other patients who have faced the same illness to learn about and manage the disease through knowledge exchange and shared experiences has proven to be an effective means of disease management (8). It necessitates interaction between the relater and the listener, facilitating the listener's ability to conceptualize and generate more valuable ideas (8–11). In this way, patients benefit from sharing their health-related experiences. This could lead to the acquisition of brand-new knowledge, methods, or abilities. The use of storytelling may be able to eliminate the stigma associated with having a disease by fostering a network of trust and equality among participants and providing an outlet for expression. Additionally, storytelling has the potential to be a vehicle for breaking resistance to health-promoting messages (12). A network of reciprocal trust and equality among participants is made possible by ST as a group activity, such as in a group of patients with a particular pathology. This reduces disease-related distress and strengthens participant relationships. It is a very helpful tool for people who struggle to share their experiences. Additionally, it enables us to learn through dialogue in the manner of the American Indian tribes' traditional teaching methods (Talking Circles), thus making it easier to communicate by speaking, asking, counting, and sharing.

Type 2 diabetes (T2D) is a widespread disease globally, both in developed and developing countries. The disease is associated with high rates of mortality and morbidity (13), such that decreasing diabetes mortality at ages younger than 25 years remains an important challenge, especially in developing countries. In addition to accompanying stress, patients with T2D may lack the ability to manage their own daily care, such as keeping a check on their blood sugar levels, adhering to a balanced diet, and taking medications when needed (14). According to Goyena and Fallis (15), patients with T2D need to engage in good self-management practices to lower their risk of complications. Being one of the most cutting-edge approaches, ST has the potential to enhance T2D patients' self-management skills and compliance with their treatment plan (2, 8–11, 16).

Storytelling (ST) is an ancient practice known in all civilizations throughout history. Traditionally, Pakistanis love telling and listening to stories (17–19). We, therefore, hypothesized that a story with a health message would be effective in the management of T2D disease (3–7). In healthcare education, the focus is on utilizing stories to provide insights into healthcare experiences, whereas in generic or managerial education settings, ST is focused primarily on communicating organizational norms and values. Therefore, it is appropriate to consider how ST can be used to convey health messages and engage service users at this point.

There is a growing body of literature that discusses the use of stories and ST as a communication tool in healthcare or health promotion; however, there are few accounts that describe how the interventions were developed, including how the end-user group provided feedback (20). While one review focused on the requirement for developmental examination, the creators remarked that this is frequently unrealistic because of restricted time and assets (20, 21). Others have noted that “developing the narrative without understanding how it is received often requires a disproportionate amount of effort” (22). The following are the goals of this study: (1) to describe the steps we took to create the story-based intervention for patients with T2D and (2) to evaluate the story using validated tools that measure the literary and scientific quality, comprehensiveness, and T2D specificity of the story. We anticipate that the information will be useful not only for future research in the field of ST but also for identifying and developing communication strategies for healthcare consumers more generally (23). We, thus, created a story with a person who experiences T2D. The main character in the story was shocked to learn that he had been diagnosed as a T2D patient. This article describes the developmental steps of health storytelling.

## 2. Materials and methods

### 2.1. Study design

This study used a mixed methodology with a predominately community-based participatory research (CBPR) design. According to Gubrium (24), CBPR is an intuitive approach to the creation of storytelling media. CBPR is a method for conducting research on health topics with community members and academia working together in an equitable partnership throughout the entire process (25, 26). The earlier study by Brooks et al. (27) served as the foundation for the current study's conceptual framework. According to Brooks et al., a purposeful way to deal with the advancement of story-based knowledge-sharing intercessions ought to include the following three stages: (1) The theory phase: The intervention's intended effects, action mechanisms, and methods for behavior change are all laid out in detail during the theory phase; (2) the modeling phase: This involves end-user participation, multiple methods, and iterative development and testing; and (3) the evaluation phase: Comprehensive formal evaluation helps ascertain whether the intervention is achieving its intended effects and adding value. The framework also provides an opportunity to enhance theory and the community's comprehension of storytelling intervention action mechanisms.

### 2.2. Ethical approval

Ethical approval for the study design was provided by the Institutional Research Board (IRB) of ISSP/NEAT (No. 012-2020). All participants gave written consent. The collection of any personal information was treated as confidential.

### 2.3. Study settings

The study was conducted in 2021 as part of a graduate student research project (A Novel Approach for Diabetes Intervention Using a Health Storytelling Technique) (28). A 10-member Research Panel (RP) was constituted by the first author (IA), which included dietitians (*n* = 4), professors of nutrition (*n* = 2), professional story writers (*n* = 3), and medical practitioners (diabetologists) (*n* = 1). RP supervised the whole process. A series of meetings and workshops were held to discuss and analyze the proceedings. The story creation part was completed in collaboration with NEAT (Nutrition, Education, Awareness, and Training), which is a registered organization of Khyber Pakhtunkhwa, Pakistan, and works in collaboration with the Integrated Social Services Program (ISSP), a KP-based not-for-profit organization. NEAT's mission is to achieve health equity by promoting health and wellbeing in the local community through education and civic engagement (29). In collaboration with ISSP/NEAT, we agreed to create a health story following standardized procedures. The purpose of the story was to serve to help patients with T2D with the correct diagnosis and treatment strategy, including how to select a balanced diet and related lifestyle habits.

**Figure 1:**
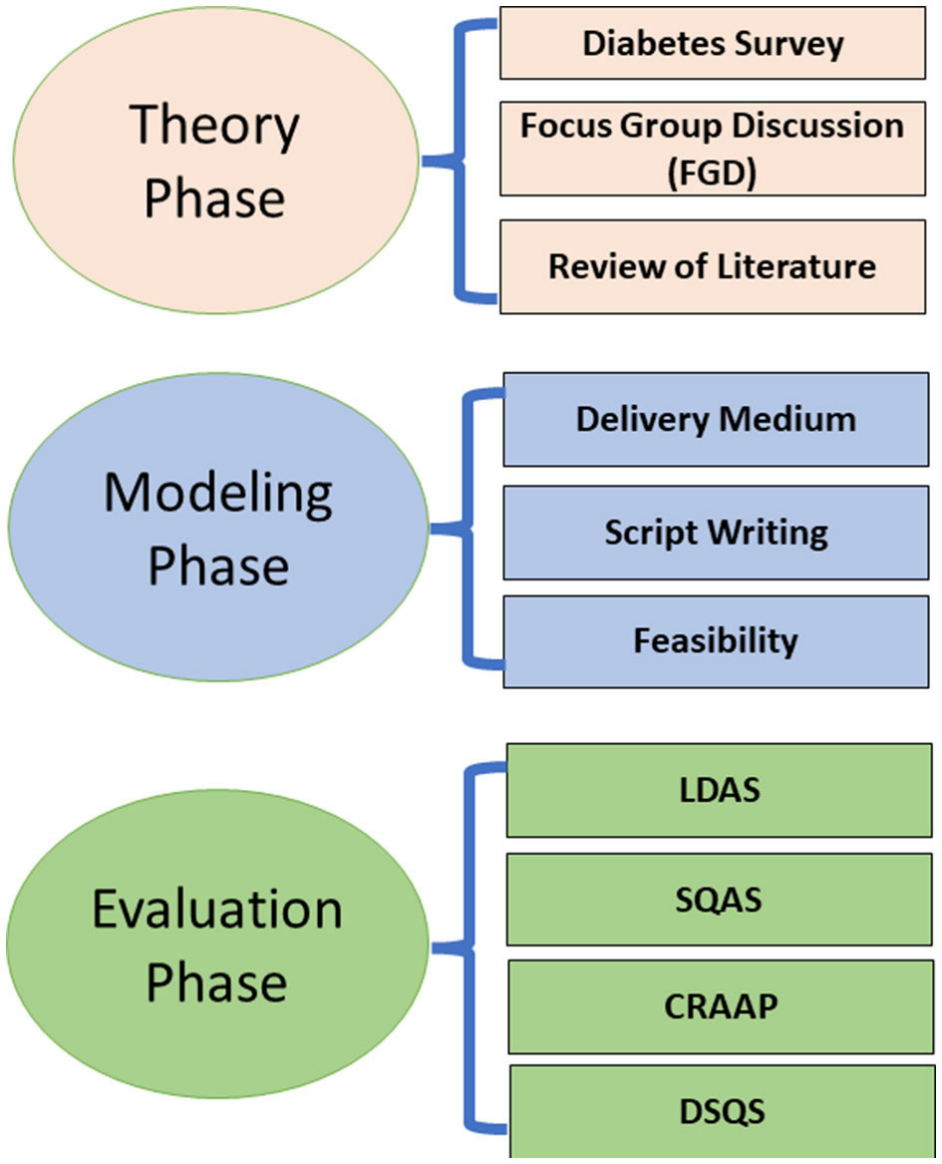
Steps involved in the development of the story.

NEAT has established a local area-based research framework and has become useful and competent by sending wellbeing-driven information through conducting health programs and results evaluation. The procedure for fostering such a network is beyond the scope of this study. However, briefly, three steps were followed for fostering a local area-based research framework. These are (1) identification of a health problem using information from various sources, including newspapers, research articles, social media networks, etc., (2) analysis of the scope of the problem (through workshops/seminars, group discussions, and surveys), and (3) devising strategies to cope with the problem. Scholarly collaborators lead each period of examination and programming together and disseminate the research results mutually at local areas, discussions, and scholastic gatherings. The experts' groups of NEAT and scholarly collaborators recognized T2D as a vital disease where intercession was earnestly required. This was additionally supported by epidemiological information showing variations in diabetes outcomes. In order to determine a strategy with the greatest likelihood of success, discussions took place regarding potential solutions drawn from personal experiences, previous partnerships, and lessons learned. The partnership adapted the ST framework. After numerous meetings and gatherings of RP members, it was decided to develop a health storytelling technique and analyze its components using scientific methods. Figure 1 shows the process and steps involved in designing and analyzing this story.

Following the framework recommended by Brooks et al. (27), the story creation was accomplished following these phases: (1) the theory phase, (2) the modeling phase, and (3) the evaluation phase. Abbreviations used: the LDAS, Literary Devices Assessment Score; SQAS, “Scientific Quality Assessment Score”; CRAAP, Assessment of Currency, Relevance, Authority, Accuracy, and Purpose); DSQS, Diabetes-Specific Questions Score.

### 2.4. Creation of diabetes story

Following the framework recommended by Brooks et al. (27), the story creation was accomplished following these phases: (1) the theory phase, (2) the modeling phase, and (3) the evaluation phase.

#### 2.4.1. Theory phase

As recommended by Brooks et al. (27), the theory phase establishes the function of intervention, that is, the means by which an intervention can change behavior. Theory is essential in identifying what to target (behavioral determinants) and how to do this (techniques to change these determinants). During the theory phase, key early tasks include (1) clearly articulating the goal(s) of the intervention and (2) developing a theoretical understanding of the likely process of change by drawing on existing evidence and theory. During this phase, (1) we proceeded to conduct a diabetes survey to evaluate community members' diabetes-related attitudes, knowledge, and practices; (2) we asked community members with T2D who participated in focus groups to inform the intervention domains; and (3) we analyzed the advancement of the intervention based on ST.

##### 2.4.1.1. Diabetes survey

In 2020, the NEAT community partners conducted surveys on 43 patients with T2D who were members of NEAT and used to pay regular visits to the NEAT clinics for weight management and nutritional counseling. Patients were identified from the membership lists of NEAT. They were contacted through emails and/or cell phones. Those willing to join the study were invited to participate in a survey followed by a Focus Group Discussion (FGD). A survey questionnaire was used using both open- and close-ended questions to know their personal experiences with diabetes, its management, medicines, food intake, and exercise, etc. The results of these surveys revealed the high burden of progressed illness and negative views (or attitudes) of diabetes and identified numerous obstacles to managing the disease. Although participants' actual knowledge of diabetes principles was relatively low, their perceived knowledge was high. Self-efficacy, high engagement in disease management, and social support from family and friends were cited as important assets by the participants. The survey, in general, provided an insight into what the overall life of a diabetic patient would look like.

##### 2.4.1.2. Focus groups discussion

Based on the findings of the survey mentioned in the previous section, an FGD was organized. All the participants in the survey were invited. However, 39 out of 43 were available for the FGD. In order to comprehend the lived and shared experiences of diabetes across four domains, the focus groups conducted on T2D patients—diabetes management, self-monitoring of glucose levels, exercise, and diet—are all important. The motivation for diabetes management and self-efficacy for engaging in the recommended health behaviors were specifically inquired about by the participants. The NEAT community and academic partners wrote a focus group moderator guide based on a literature review and consensus. The questions were formulated in a phenomenological manner to elicit the lived experience of diabetes in four distinct domains. Digital recordings of the focus groups and post-focus group debriefings were then translated into English and transcribed. Each discussion in the focus group was recorded by a notetaker who used the same language. A total of seven FGDs, with six people participating in each, were held. The present study's participants demonstrated significant information needs regarding diabetes and the prescribed treatment. The main obstacles to medication and/or diet adherence were the characteristics of the regimen (such as the route of administration, number of prescribed medications, dosage frequency, and perceived side effects), low knowledge about balanced diet, glycemic index of foods, mealtimes, meal-type suitability with mealtime, etc. Participants showed high stress due to T2D, fear of related diseases, social stigma, and self-perceived social pressure. Participants not only displayed negative attitudes and low self-efficacy to adhere to necessary self-care activities such as diet, physical activity, and self-monitoring of blood glucose, but they also demonstrated negative beliefs about the illness and the medications that were prescribed to them. Moreover, there was a common belief in the efficacy of alternative medicines and other common misbeliefs. The details of the methodology and themes derived from these FGDs are provided in Supplementary material S1.

##### 2.4.1.3. Review of literature

Information extracted from a diabetic survey and FGD was written on paper for a meeting of the RG to analyze and discuss them further. There was a common consensus among the members of RG regarding the authenticity of the information extracted. However, for more clarity, a thorough review of the literature was suggested to prepare a list of topics to be included in the story during the modeling phase that follows in the next section.

#### 2.4.2. Modeling phase

The core of narrating knowledge-transferring mediations exists in the modeling phase: how to construct and present a compelling narrative that has the potential to motivate behavior change while preserving and emphasizing an evidence-based message. The modeling phase involves writing the story script and choosing the ST method after establishing the intervention's purpose and the science behind the techniques for changing behavior. In order to apply science to the specific contexts in which the intervention will be implemented, this phase necessitates artistic ability. In this phase, we decided on the delivery medium and script writing.

##### 2.4.2.1. Delivery medium

As noted by Brooks et al. (27), the decision of delivery medium is one of the critical decisions to make for a story to effectively communicate knowledge sharing. Based on the observations of the research panel (RP), a thorough literature review (30), and comments from the patients who participated in the survey and the FGD, we decided to present the story in the form of a book (written text) as it is the easiest and most feasible way for the community members to access or communicate with.

##### 2.4.2.2. Story script

As noted by Brooks et al. (27), stories are frequently depicted as conventional (for example, setting everything up, laying out the subject, presenting the plot, and coming to a goal). In any case, it is more exact to depict this as the fundamental type of story, as narrating is an art with no recipe to produce positive messages that will reverberate with clients. A story form can be complicated and require many parts. An “abstract” (what the story is about), an “orientation,” a “complicating action,” an “evaluation,” a “resolution,” and a “coda” (or what serves as a link between the narrative and the “present situation”). However, a compelling narrative can also be as simple as a single line or even an image in which an outcome can be deduced from a complication. Keeping these requirements in consideration, we partnered with local story writers (SWs: *n* = 3). These SWs had more than 30 years of experience in story writing. They were provided with the notes and list of topics generated in the previous phase. They were asked to complete an initial draft of the story within 2 weeks' time with these tasks: (1) Oral story sharing and transcription; (2) script writing from the initial transcript; (3) story script editing; (4) story voiceovers; (5) collection of images; (6) sub-titling; (7) story production; (8) editing; (9) approval of the final product. They were asked to work in a group. After the stipulated time, a first draft of the story was received and analyzed in a meeting of the RG, accompanied by the SWs. Some minor changes in phrases were incorporated, and at some points, some corrections were made. For example, nutritional supplements and medicines are usually mixed up in the local community. We, therefore, made corrections at places to think of them as two distinct things. Similarly, the words used for “constipation” and “diarrhea” in the local language are usually confused, and therefore, whenever these are used in the story, a small description or associated symptom accompanies each term to clearly differentiate between the two.

##### 2.4.2.3. Feasibility

To ensure the intervention's viability, we piloted the prototype in accordance with the MRC's recommendations. This feasibility testing—also known as usability testing—is a crucial first step in ensuring that the ST intervention will address the modeling difficulties and have the intended effects on behavior change. scientists take risk in preparation and informing entanglements that might make the mediation come up short (31). For the present study, to test the feasibility, we used the “Recall” method, where the story was narrated to a group of 10 people (5/5 male/female), and recall was measured by asking them what they remembered about the story (31). Their recall was assessed through 20 questions focused on the themes of stories. The results showed excellent recall (mean 90% correct answers; range 85–100%), demonstrating a high feasibility (Supplementary material S2).

As a final step, we also obtained feedback on the overall quality (easiness, understandability, correctness of information, etc.) of the patient participants of the FDG and those of the feasibility recall session. This feedback was important and useful in the final editing of the story script.

#### 2.4.3. Evaluation phase

Evaluating the ST intervention following its pilot testing, modeling, and implementation is a necessary phase before the story may be used on a community basis. Therefore, the story developed during the modeling phase was subjected to an array of quality analysis tools. The quality analysis of the story was conducted for four main purposes: (1) the “Literary Devices Assessment Score” (LDAS), (2) the “Scientific Quality Assessment Score (SQAS), (3) CRAAP (assessment of Currency, Relevance, Authority, Accuracy, and Purpose), and (4) Diabetes-Specific Questions Score (DSQS). Three out of four scales (LDAS, SQAS, and DSQS) were developed by our group for the purpose of the present study. These scales were developed and validated, and the results are pending publication. In general, the item-level content validity index (I-CVI) and scale-level content validity index (S-CVI) were found to be 80, 80, and 85%, respectively, for LDAS, SQAS, and DSQS. See a summary of the validation studies in Supplementary material S3. Cronbach's alpha score for these scales was >0.8, suggesting a satisfactory coefficient of reliability. The other scale (CRAAP) is a well-validated score previously used in research studies (32–34). We have vast experience in scale development and validation and have developed scales in the past (35–37).

***LDAS:*** For the literary quality assessment, a pre-developed and validated questionnaire was used for data collection on the quality parameters of the story, including these 15 components. The 15 components that make LDAS are as follows: “allusion,” “diction,” “alliteration,” “allegory,” “colloquialism,” “euphemism,” “flashbacks,” “foreshadowing,” “imagery,” “juxtaposition,” “metaphor/simile,” “personification,” “onomatopoeia,” “symbolism,” and “tone.” The story scored 0–3 points. Every classification is weighed similarly. On this scale, 45 points is the highest possible total score. If the final score is between 0 and 8, it probably comes from a questionable or unreliable source of information. Information may be reliable with a score between 9 and 17, but caution should be exercised. A score between 18 and 36 indicates a reliable information source, and scores between 37 and 45 indicate an excellent information source.

***SQAT:*** We utilized the 26-item Scientific Quality Assessment Test (SQAT) scale for evaluating “scientific quality.” There were 26 items with scores ranging from 0 to 3. The 24 components that make SQAT are as follows: “Verified Evidence,” “Direct Evidence,” “Unbiased Evidence,” “Evidence Within Context,” “Independent Source,” “Multiple Sources,” “Verified Source,” “Authoritative/Informed,” “Known Sources,” “Within Context Sources,” “Objectivity of the Sources,” “Source Attribution,” “WHO Guidelines,” “Nutrition Advice,” “Physical Activity Advice,” “Medication Advice,” “Lifestyle Advice,” “No Conflict of Interest,” “Cost Effective,” “Benefits Quantified,” “Side-effects Declared,” “Alternativity,” “Availability,” and “Novelty.” All components were weighed similarly. The highest possible total score is 78 points. If the final score is between 0 and 19, it probably comes from a questionable or unreliable source of information. Information may be trustworthy with a score between 20 and 39, but caution should be exercised. A score between 40 and 59 indicates a reliable information source, and scores between 60 and 78 indicate an excellent information source.

***CRAAP:*** Sarah Blakeslee and her team of librarians at California State University created the CRAAP (Currency, Relevance, Authority, Accuracy, and Purpose) test (38), which is used to evaluate the content's reliability. The CRAAP test has been utilized both as a showing device in scholastic settings and as an objective technique to assess information sources in other different clinical examinations (38). The details of CRAAP are provided in Supplementary material S3. Briefly, the five components that make CRAAP are “Currency,” “Relevance,” “Authority,” “Accuracy,” and “Purpose.” The components received a score between 0 and 3 points based on the criteria for each of the five CRAAP components (see Supplementary material S3). Every component was weighed similarly. The maximum possible total score is 15. A final score of 0 to 3 points indicates a questionable and probably unreliable source of information. Information may be reliable with a score between 4 and 7, but caution should be exercised. A score between 8 and 11 indicates a reliable information source, and scores between 12 and 15 indicate an excellent information source.

***DSQS:*** The Diabetes-Specific Question Score (DSQS) is a novel instrument designed to objectively evaluate diet-related content that is relevant to people with T2D in the present story. A panel of diabetologists, clinical dieticians, and nutritionists with expertise in the nutritional management of diabetic patients reviewed the DSQS tool before it was developed by the researchers of this study. Fifteen questions on the main nutritional aspects of diabetes make up the test. The details of DSQS are provided in Supplementary material S3. Briefly, the 15 components that make DSQS are as follows: “Nutritional Causes,” “Signs and Symptoms,” “Medical and Nutritional Care,” “Diagnosis-How,” “Diagnosis-Blood Sugar level,” “Nutritional Management-When,” “Nutritional Management-How,” “Nutritional Management-Carbohydrates,” “Nutritional Management-Elimination of Sugar,” “Nutritional Management-High Protein,” “Nutritional Management-Consistency in Carbohydrate,” “Nutritional Management-Supplements,” “Nutritional Complications,” “Nutritional Complications-Ketoacidosis,” and “Nutritional Complications–Insulin.” Each question received a score between 0 and 3 points (Supplementary material S3). On this scale, 45 points is the highest possible total score. If the final score is between 0 and 8, it probably comes from a questionable or unreliable source of information. Information may be reliable with a score between 9 and 17, but caution should be exercised. A score between 18 and 36 indicates a reliable information source, and scores between 37 and 45 indicate an excellent information source.

### 2.5. Data analysis

In line with some previous studies by Stein et al. (39), Liu et al. (40), and Omidbakhsh (41), the health story was assessed by four raters–two raters specializing in nutritional and general care in diabetes evaluated the story for its nutritional knowledge contents, while the remaining two raters, who were experts in literature, evaluated the literary quality of the story. As a prerequisite, the raters selected had at least a Ph.D. in nutrition/health (for the first two raters) or a Ph.D. in drama script writing with at least 5 years of teaching/research experience in addition to their past experience as an author/reviewer of research studies of the same nature. The raters rated the story independently of each other using validated rating tools with established criteria as “extremely satisfactory” (ES), “satisfactory” (S), “unsatisfactory” (US), or “not applicable” (NA) in each criterion. For each criterion in the tools, the statements were scored as “extremely satisfactory” (2), “satisfactory” (1), “not satisfactory” (0), or “not applicable” (no score) if the statement was not relevant. According to a coding guide, scores were also expressed as percentages of the total assessable items deemed extremely satisfactory or satisfactory. To resolve any score differences between raters, regular face-to-face meetings were scheduled. In this study, we identified the following types of scores: a satisfactory score for the individual, a satisfactory score for the group, a satisfactory score for the criterion, and an average satisfactory score. Regarding the interpretation of the evaluation, the raters came to an agreement. Information on examination for the review incorporated the computation of the kappa coefficient and the Pearson Item Second relationship coefficient to evaluate the rater understanding between two autonomous scientists. The mean values, percent (%), number of units (n), and standard deviation (SD) were reported as applicable for descriptive statistics. All tests were deemed statistically significant when the *p-*value was <0.05, and agreement for kappa coefficients was evaluated using Landis and Koch (42) criteria. SPSS version 28.0 was used for every single analysis of the data. The NEAT Institutional Review Board granted ethical approval, and all participants, including raters, gave written consent to participate in the study.

## 3. Results

We generated a story with the title *da sugar qeesa–qeesa da kamyabi* (in English: “The Story of Diabetes–the Story of Success”). This 86-page story is in the local language, Pashto, and has also been translated into English. The final story was compiled in the form of a standard book with a cover and an illustration depicting the main themes of the story in a graphical form. A summary of the story in the original language (“Pashto”) with English translation is given in the Supplementary material S4.

Briefly, the story tells how the main character, a diabetic patient, coped successfully with the situation by adhering to self-care and a balanced diet. The full story text with an English summary is provided in the Supplementary material. However, briefly, the story is divided into four chapters. The first chapter (titled: “*From ‘tingling' feet to eye injections”*) describes how the main character of the story started observing some initial symptoms. “*……a mild tingling in feet, or ‘pins and needles', was the first diabetes symptom he [Javed: the main character] experienced around some 5 years ago. Javed had been unwell for some time, and despite taking medication from a doctor, his condition did not improve. He at times had the feeling that it was deteriorating. He fell on the ground 1 day, and when he was taken to the medical clinic, he figured out that the issue had not been as expected analyzed. He was being given medication by the specialist and was seeing without having any tests done in the lab. The doctors at the hospital were worried that the drugs might have hurt more than helped, and that the so-called doctor might not even have been a good doctor but just lied. This deteriorated over the long run and at last prompted deadness in a portion of his feet.”* The second chapter (titled *Caught Among the Healers*) describes how he got confused among unregistered quack healers, including local practitioners, *Hakeem, homeopaths*, and even magicians and spellers. Owing to wrong investigations and medication as a follow-up, his condition worsened. The symptoms became more severe. The third chapter (titled “*Coming Across a Diabetic Club”*) describes how he visited a diabetic club and met its members, including a few diabetologists and dietitians. The chapter narrates how he was advised to get his fasting blood glucose level and other necessary tests, including a glucose tolerance test and HBA1c test. Chapter 4 (titled “*The Grief to be Diagnosed as a Diabetic”*) describes how terrifying it was to know that he had type 2 diabetes, as confirmed by the tests mentioned in Chapter 3. The chapter also describes how he again was a victim of the same “quakes” for easy medication and treatment. The final chapter (titled: “*Again Toward the Diabetic Club”*) describes how his family and friends guided him to go to the diabetic club to learn the required changes in diet and lifestyle to cope in a better way with the disease. The chapter details how he benefited from the experiences of diabetic patients of the club regarding adopting balanced dietary habits, exercise, and other related lifestyle changes. The chapter also details the interesting cooking classes arranged by the club and the healthy meal recipes by the dietitians. The chapter ends with a TV interview from a local channel asking him (the main character) how successfully he coped with the disease by adopting a balanced diet and healthy lifestyle habits. Through this interview, he gave a detailed note on the importance of eating vegetables, citrus fruits, fiber, plant protein, fish, milk, etc., and the negative effects of simple sugars, sweets, etc.

The results of the content evaluation show that the health story received a mean total score of 25.4 out of a possible total of 45 on the LDAS, as shown in Table 1, indicating a high level of literary quality. The story's scientific quality score, as determined by SQAT, is shown in Table 2. From a total of 78, the mean total SQAT score was 41.3. In terms of inter-rater reliability, LDAS and SQAT had Pearson correlations of 0.83 and 0.81, respectively. Pearson correlation scores of >0.81 in accordance with the generally accepted Landis and Koch (42) criteria indicate excellent agreement between the two raters. The story received a mean CRAPP score of 8.86 out of a possible 15 points, which is a good source of information but does not meet academic standards (Table 3). As shown in Table 4, the mean DSQS total score was 28.2 out of an overall possible score of 45, which is considered to be “fair.” Between-rater dependability scoring showed a Pearson correlation of 0.80 and 0.85 for the CRAAP and DSQS all-out scores, respectively.

**Table 1:** LDAS^*^ mean scores with interrater reliability.

**Allusion**	**Mean score**	**Kappa**	**Pearson correlation**	***P-value***
Diction	1.8 ± 0.50	0.75		<0.001
Alliteration	1.5 ± 0.70	0.65		<0.001
Allegory	1.8 ± 0.55	0.86		<0.001
Colloquialism	1.5 ± 0.70	0.81		<0.001
Euphemism	1.7 ± 0.90			<0.001
Flashbacks	1.8 ± 0.80	0.75		<0.001
Foreshadowing	1.8 ± 0.60	0.65		<0.001
Imagery	1.6 ± 0.60	0.86		<0.001
Juxtaposition	1.8 ± 0.50	0.81		<0.001
Metaphor/simile	1.5 ± 0.40			<0.001
Personification	1.8 ± 0.50	0.65		<0.001
Onomatopoeia	1.5 ± 0.60	0.55		<0.001
Symbolism	1.7 ± 0.60	0.65		<0.001
Tone	1.8 ± 0.60	0.75		<0.001
**Total score**	**25.4** **±6.45**		**0.83**	

^*^LDAS, Literary Devices Assessment Score; *p*-value reflects significance of interrater reliability score; *p* <0.05.

**Table 2:** SQAS^*^ mean scores with interrater reliability.

**Scientific quality items**	**Mean score**	**Kappa**	**Pearson correlation**	***P*-Value**
Verified evidence	1.6 ± 0.65	0.75		<0.001
Direct evidence	1.8 ± 0.65	0.65		<0.001
Unbiased evidence	1.8 ± 0.70	0.86		<0.001
Within context evidence	1.5 ± 0,76	0.81		<0.001
Independent source	1.5 ± 0.75	0.55		<0.001
Multiple sources	1.7 ± 0.80	0.65		<0.001
Verified source	1.8 ± 1.10	0.75		<0.001
Authoritative/informed	1.8 ± 0.85	0.75		<0.001
Known sources	1.6 ± 0.75	0.68		<0.001
Within context sources	1.8 ± 0.81	0.69		<0.001
Objectivity of the sources	1.5 ± 0.73	0.65		<0.001
Source attribution	1.8 ± 0.55	0.65		<0.001
Who guidelines	1.7 ± 0.75	0.55		<0.001
Nutrition advice	1.8 ± 0.71	0.57		<0.001
Physical activity advice	1.8 ± 0.65	0.65		<0.001
Medication advice	1.6 ± 0.87	0.65		<0.001
Lifestyle advice	1.8 ± 0.86	0.65		<0.001
No conflict of interest	1.8 ± 0.74	0.55		<0.001
Cost effective	1.7 ± 0.73	0.55		<0.001
Benefits quantified	1.8 ± 0.83	0.65		<0.001
Side-effects declared	1.8 ± 0.56	0.55		<0.001
Alternativity	1.8 ± 0.85	0.55		<0.001
Availability	1.8 ± 0.44	0.57		<0.001
Novelty	1.7 ± 0.76	0.65		<0.001
**Total Score**	**41.3** **±** 12.2		**0.81**	

^*^SQAS, Scientific Quality Assessment Score; *p-*value reflects significance of interrater reliability score; *p* <0.05.

**Table 3:** CRAAP^*^ mean scores with interrater reliability.

**Items**	**Mean score ±SD**	**Kappa**	**Pearson correlation**	***P-*value**
Currency	1.89 ± 0.70	0.72		<0.001
Relevance	1.46 ± 0.65	0.58		<0.001
Authority	1.91 ± 1.20	0.59		<0.001
Accuracy	1.77 ± 1.30	0.35		<0.001
Purpose	1.79 ± 1.50	0.40		<0.001
**Total score**	**8.86** **±3.16**		**0.82**	**<0.001**

^*^CRAAP, Currency, Relevance, Authority, Accuracy, and Purpose; *p*-value reflects significance of interrater reliability score; *p* <0.05.

**Table 4:** DSQS^*^ mean scores with interrater reliability.

**DSQS test**	**Mean score ±SD**	**Kappa**	**Pearson correlation**	***P-*value**
Nutritional causes	1.85 ± 0.75	0.65		<0.001
Signs and symptoms	1.85 ± 0.65	0.59		<0.001
Medical and nutritional care	1.90 ± 0.66	0.50		<0.001
Diagnosis-how	1.70 ± 0.78	0.50		<0.001
Diagnosis-blood sugar level	1.75 ± 0.43	0.45		<0.001
Nutritional management-when	1.85 ± 0.65	0.65		<0.001
Nutritional management-how	1.80 ± 0.54	0.59		<0.001
Nutritional management-carbohydrates	1.85 ± 0.65	0.50		<0.001
Nutritional management-elimination of sugar	1.80 ± 0.75	0.50		<0.001
Nutritional management-high protein	1.90 ± 0.88	0.65		<0.001
Nutritional management-consistency in carbohydrate	1.85 ± 0.76	0.59		<0.001
Nutritional management-supplements	1.75 ± 0.75	0.50		<0.001
Nutritional complications	1.65 ± 0.65	0.50		<0.001
Nutritional complications-ketoacidosis	1.70 ± 0.75	0.45		<0.001
Nutritional complications-ketoacidosis	1.65 ± 0.75	0.65		<0.001
Nutritional complications- insulin	1.55 ± 0.80	0.59		<0.001
**Total score**	**28.40** **±7.41**		**0.85**	

^*^DSQS, Diabetes-Specific Question Score.

## 4. Discussion

The story was developed with the purpose of educating patients with T2D on diet, lifestyle, and other related medical issues. Story evaluation and analysis demonstrated higher total mean values for all scores used in the study. All tools were validated and discussed with the experts in the field. Besides the fact that a story should be sound regarding its contents against the updated modern-day scientific facts, it should at the same time be engaging enough to attract and impact the readers. In this way, health-related messages can be conveyed more effectively across a wide range of the target population. As previously suggested, storytelling is a potentially beneficial training modality and practicable for the self-management behaviors of adolescents with type 1 diabetes (4) and for health promotion interventions among T2D (3). Therefore, the story developed in this study was validated by utilizing certain tools to assess its scientific and literary attributes. The LDAS, SQAS, and DSQS criteria could be used as a grading tool to evaluate the quality of educational content for other diseases in future studies if they are validated in more generalized forms. The same parts and questions of the LDAS, SQAS, and DSQS criteria, for instance, could be studied in the context of stories about cancer or hypertension. Inter-rater reliability scoring showed a Pearson correlation of 0.83, 0.81, 0.82, and 0.85 for the LDAS, SQAS, CRAAP, and DSQS total scores, respectively. Per the commonly accepted Landis criteria, Pearson correlation scores of >0.81 demonstrate excellent agreement among the raters (42).

The main purpose of the current study was to develop a high-quality health story based on the current scientific evidence related to T2D. Therefore, for the present study, assimilation was given core consideration while developing the story intervention. The local population traditionally has a strong belief in storytelling and story listening (18). The study intervention was developed based on the successfully used protocols developed for the reviewing, editing, and packaging of storytelling. However, all content for ST interventions is stored locally, based on Pakistani Pashtun ethnic participants who speak naturally about their situation with their personal and cultural beliefs. With this objective, we developed a story and assessed its quality against an established set of parameters (24, 25). This strategy empowers communities, raises awareness of issues that are culturally relevant, and targets numerous obstacles to health. A previous study indicated that the Virtual Diabetician does possess a set of components that can efficiently provide diabetes information to patients using innovative storytelling-based information (6). In this study, we developed an ST intervention to reduce diabetes-related health disparities in T2D patients, building on an established partnership with CBPR. We describe the process and outcomes of the participatory development of a diabetes ST, as well as the focus group results regarding the lived experience of diabetes among these populations. The formal act of telling a story about a real-life experience is known as storytelling.

According to Roy (43), an author might, for instance, relate a life experience that inspired them. The author clearly describes the experience in this instance. In addition, the individual may include any other characters or objects that are in the encounter. In essence, a narrative should define a story about main characters who participate in a variety of events. Primary characters in a story participate in invigorating, critical, or engaging encounters. Consequently, individuals give a brief depiction of occasions in story writing to draw in the consideration of the crowd. Creative narratives pique the interest of the reader and encourage them to continue reading. According to Roy (43), one example is when a writer chooses a story topic or experience to tell; a narrative's heading and introduction ought to be appealing in this instance. In addition, the narrative's structure ought to have a smooth flow of ideas. Therefore, a writer's level of creativity determines its quality. A story's opinions and tone should demonstrate the author's individuality. Creative writing, therefore, relies on original concepts. Through imagination, a creator of a story advances to utilize uncommon shows, word decisions, and sentence familiarity (43).

For story development, we followed a careful and broad cycle to create a story with the main goal of creating an intervention that could be used in a randomized controlled trial to investigate how well ST might work as a communication tool. We also found that involving the end-user group in the development of such an intervention provided the best guidance for a number of decisions. A repetitive theme of the feedback from the FGD was the capacity of the story to connect with the story and relate to the characters, which has been recognized as a vital variable for stories to be successful (21, 44). For accuracy and to tailor the storybook to the requirements and preferences of the end users, specific feedback regarding the story's content, errors and inconsistencies, and presentation style was essential.

Story writing for health communication purposes is not that simple because a health story is much different from a fiction story. There are many challenges one may face while developing a story for health communication (20). A big challenge we had while compiling the story was staying true to the story vs. being evidence based (20). For example, at one place in the story, Javed (the main character) had been misdiagnosed even by a registered physician. We had to consider whether it was appropriate to point out that physicians may make errors in diagnosis. We decided to include the incident in the story to highlight a relatively common error of misdiagnosing the disease. It also provided an opportunity in the story to educate the patients about the differences in disease types (type 1 vs. type 2) and particularly the different treatments appropriate for each type. Another predicament in regard to being evidence-based was the decision about whether to deploy nutritional supplements for which there was no evidence. For example, there is far and wide practice and suggestions around the utilization of certain foods (particularly vegetables) and vitamins regardless of no exploration proof supporting their viability. The use of drug names and the possibility of product placement posed another problem. For instance, many patients would be more acquainted with a particular drug or supplement package as compared to some other one. We decided to use trade names that are more familiar to laypeople, but we also included a variety of product names so that we would not appear to favor any one brand.

The amount of additional information or evidence to include in the story was another related issue (20). Many reviewers (patients and members of the RP) in the present study desired more in-depth medical data. However, the patients' accounts of the events rarely considered this. Eventually, we incorporated a decent lot of data about T2D and its administration (e.g., signs and side effects, what insulin is, how the medications are managed, how meals and nutrients can affect blood glucose, glycemic index of foods, etc.), yet we attempted to integrate this detail as flawlessly as possible into the story while saving its story stream and tone. Additionally, we felt obligated to provide appropriate information regarding when to seek emergency or additional care. Creating stories that are appealing and easily generalizable was a major challenge (20, 45). The length of the story, the reading level, the narrative mode (e.g., first person or third person), the representation of various demographics (e.g., sex, race, age, and socioeconomic status), and the representation of various illness experiences (e.g., severity of illness, hospitalization, and management at home) were just a few of the many considerations that came into play (20). To increase the likelihood that patients would read the entire story, we had to strike a delicate balance between being as inclusive, generalizable, and detailed as possible (20, 44) and being as succinct as possible (20, 21, 46). The end-user group, specifically those who are most likely to benefit from the intervention, needs to be the primary source of information for these considerations. The story presentation expenditure of resources was also a major consideration. Stories can be told in a variety of ways, including computer games, video games, cartoons, and so on. We had already decided to use a paper-based story format for this project. However, there were still a lot of things to think about in this format, such as the kind of illustrations, how graphics and color were used, and the size and shape of the book (20). Again, the information we got from the patients' focus groups helped us make decisions. The preferences of the intended audience determined the best way to present the story. Our process was constrained by the limited number of individuals we were able to recruit for our focus groups despite the rich and instructive feedback we received. We put out a lot of ads in a lot of places, but very few people agreed to take part. We had a particularly difficult time recruiting because our target audience consisted of parents with young children who had many competing priorities and limited time.

The main strength of the present study is that we followed a systematic way to create a story that is for educational purposes for diabetic patients. The framework of Brooks et al. (27) was followed for story development. The tools used for story evaluation were all validated. There are also some limitations of the study. First, it was carried out within a single community, which may limit its applicability to other T2D patients. Second, only one story was created, which is a little arbitrary, even though other studies aimed at creating stories that are culturally relevant to influence health behaviors (47) influenced the decision. Future research ought to focus on how many stories are required to deliver a sufficient dose that addresses each behavioral construct and motivational principle in the shortest amount of time. Third, this study does not demonstrate intervention effectiveness. However, the story was used on a limited number of T2D patients in a separate study [data not shown in this manuscript and are under consideration in a separate manuscript by Ali and Alam (28)]. Nevertheless, testing the effect of the intervention on the diabetes process and outcome measures in clinical settings will be the primary focus of subsequent research.

Patients can be educated in a unique way through healthy ST. Policymakers and healthcare administrators should think about using storytelling materials with catchy titles to meet the needs of diabetic patients and the public to reduce the amount of misinformation and gaps in the facts. It is essential for healthcare providers to comprehend the characteristics of these stories and be aware of potential sources of misinformation due to the frequency with which patients utilize story traditions for healthcare information. Interventions that encourage people with diabetes to tell their own stories and allow them to speak in their own words are called storytelling. Culturally tailored stories have been used to change health behaviors among ethnic minority populations as an intervention (48–50). Every effort will be made to make it possible that the storybook developed may be freely available, both in hard and soft forms, to the people with diabetes who were not part of the present intervention.

## 5. Conclusion

In conclusion, our study demonstrated that a story related to nutrition and self-care of diabetic patients has a wide range of quality, accuracy, and purpose. The innovative health ST developed for T2D was assessed and found to be of significant satisfaction with respect to literary quality and scientific soundness and is recommended to be used for further studies for the better management of T2D.

## Data availability statement

The original contributions presented in the study are included in the article/Supplementary material, further inquiries can be directed to the corresponding authors.

## Ethics statement

The studies involving humans were approved by Ethical Approval for the study design was provided by the Institutional Research Board (IRB) of Nutrition, Education, Awareness and Training under ISSP/NEAT (No. 012-2020). The studies were conducted in accordance with the local legislation and institutional requirements. The participants provided their written informed consent to participate in this study.

## Author contributions

SAli, SB, AU, IA, and FZ initiated and designed the study. SAli, SB, and AU collected the field data under the supervision of IA, AA, BS, and FZ. IA, FZ, and AU conducted the survey and focus group discussion. TO, RO, MF, LC, FN, HR, HH, MH, SAlB, and BS performed the statistical analysis and wrote the first draft of the manuscript. IA and FZ supervised the study and acted as guarantors. All authors contributed to the critical revision and editing of the manuscript.
