# Erratum: An innovative state-of-the-art health storytelling technique for better management of type 2 diabetes

**DOI:** 10.3389/fpubh.2024.1386189

**Published:** 2024-03-18

**Authors:** Frontiers Production Office

**Affiliations:** Frontiers Media SA, Lausanne, Switzerland

**Keywords:** storytelling technique, type 2 diabetes, health education, innovative health techniques, health story

Due to a production error, the affiliation number for the author MoezAllslam Ezzat Faris was incorrectly written as “3,4” instead of “4,5”.

The publisher apologizes for this mistake. The original article has been updated.

