# Supplementary material for: An innovative state-of-the-art health storytelling technique for better management of type 2 diabetes
Source: Front Public Health. 2023 Sep 29;11:1215166. doi: 10.3389/fpubh.2023.1215166 (PMC10585594; doi:10.3389/fpubh.2023.1215166)
Supplement: Supplementary file 1 [file Data_Sheet_1.PDF]

Annexure I

Letter: Dr. Aftikhar Alam

The Story of Diabetese – the Story of Success

Although diabetes is not a completely cureable disease - this disease cannot be prevented by treatment - diet is a treatment for this disease with good diet, a diabetic patient - can live an almost normal life - bad diet is an enemy for this disease. - In order for the diabetic patient to be aware of this bad diet, it is necessary to act on the advice of the .doctors

It is very important to go to the doctor for an examination from time to time and it is also very important to take a sugar test - so that the .patient is aware of the sugar level

In addition to doctors, there are organizations that publish small booklets on the use of various foods

Annexure I

لیک: ډاکټرافتخار عالم

د شوګر رنځ او د هغهٔ مخ نیوے

د شوګر مرض کهٔ هر څو یو ښهٔ مرض نهٔ دے- په علاجونو هم دا مرض دفع کیږي نه – خوپرهېز ددې مرض دپاره دعلاج حیثیت لري- په ښهٔ پرهېز سره د شوګر مریض تقریباً یو نارمل ژوند تیېرولے شي- بدپرهېزي ددې مرض دپاره د یو دشمن حېثیت لري- ددې دپاره چې د شوګر مریض ددې بدپرهېزی نه خبر شي د ډاکټرانو په مشوره عمل پکار دے-

بل وخت په وخت د معاینې دپاره ډاکټر ته تلل ډېر ضرور دي او ورسره د شوګر ټې سټ اېستل هم ډېر ضروري دي-چې مریض د شوګر لېول نه خبر وي-

د ډاکټرانو نه علاوه داسې ادارې هم شته چې د شوګرمریض دپاره د مختلفو خوراکونو د استعمال په لړکښې وړې وړې کتابچې شائع کوي- هغه دځان سره ساتل هم ډېر ضروري دي-

for diabetics - it is very important .to keep them with you

It is very important for a diabetic patient to protect himself from drugs, which can become a cause .of harm instead of benefit

The mentioned book has also been written on these topics, in which all these problems and their solutions .have been presented in great detail

This book is the story of a servant named Javed, who lived a very happy and prosperous life with his wife- serving in a big chair in a large private company- because of his ability and competence. He had the opportunity to travel to European countries several times - because Javed was successful and successful in this journey of life - but suddenly his life changed its course instead of bright and happy. Kapro - and Dashto went to the past - in this journey of success, Javed appeared tired and bored - he would not enjoy going to the office, but he would not be able to do any work in the office he would always be in his thoughts. -

د شوګر مریض دپاره دا ډېره ضروري ده چې د ټوټکو نه ځان وژغوري دا د فایدې په ځائے د نقصان وجه ګرځېدے شي-

ذکر کوونکے کتاب هم په دې موضو لیکلے شوے دے چې پکښې دا ټولې ستونزې او ددې حل ډېر په تفصیل سره وړاندې کړے شوے دے-

دا کتاب د یو داسې بنده چې دا جاوېد په نامه نومیږي قیصه ده چې د خپلټبر سره ډېر دخوشحالو او سوکالو ژوند کولو- په یوه پرائېوېټ لویه کمپنۍ کښې په یوه غټۀ څوکۍ ناست خپل خدمات تر سره کول- د خپل قابلیت او صلاحیت په وجه ورته څو څو ځله اروپائي هېوادونو ته د سفر کولو موقعې هم په لاس ورغلې وې- غرض دا چې جاوېد د ژوند په دې سفر کښې ښۀ کامیاب او کامران روان ؤ- خو اچانک د هغۀ ژوند خپل روخ بدل کړو- د رڼاګانو او خوشحالو په ځائې کندو کپرو او د شتو تیرو ته روان شو- د کامیابۍ په دې مزل کښې جاوېد ته ځان ستړے ستومانه ښکاره شو- هغۀ له به نۀ دفتر ته تلل خوند ورکؤل بلکې په دفتر کښې به ترې هېڅ قسمه کار هم نۀ کېدو- هغه به هروخت په سوچونو کښې ډوب ؤ چې په ما باندې اخر څۀ وشو او دا څۀ راسره روان دي کیږي-

قیصه هم دغسې مخ په وړاندې روانه وه چې یوه ورځ د جاوېد په پښه کښې زخم

I was shocked at what happened to me .and what is happening to me

The story was going forward like that, one day Javed got a wound on his foot and he didn't take the name of the injury - Javed wanted to meet the doctor

After the examination, the doctor told Javed that you are suffering from diabetes - Javed's voice reached him, so he shot a gun - he was never ready to accept that he was diagnosed with diabetes. hit

:Deceiving yourself

Javed never believes that sugarcane is my sugar - his own friend Afzal also advises him that all doctors do it all for money - and they lie - he takes Javed to a homeopathic doctor - but Javed is also disappointed by them - because Javed is an educated and experienced person, that is why he goes to various inexperienced doctors and sages on the advice of his relatives and friends - but he never follows their advice. He does and does not use medicines - this will worsen his condition - one day he may be

راغلو او د جوړېدو نوم ئې نه اخستو- جاوېدډاکټر سره د مېلاوېدو اراده وکړه-

ډاکټر د معاينې نه پس جاوېد ته ووئېل چې تاسوته د شوګر مرض لګېدلے دے- د جاوېد چې دا آواز تر عوږو شو نو لکه چې چاپرې د ټوپک ډز وکړو- هغه دې قبلولو ته هېڅ کله هم تيار نۀ ؤ چې ګني هغۀ ته د شوګر مرض لګېدلے دے-

ځان په دهوکه کښې ساتل:

د جاوېد هېڅ کله هم پدې يقين نۀ رازي چې ګني زما شوګر دے- د هغۀ خپل دوست افضل هم هغۀ ته دا مشوره ورکوي چې ټول ډاکټران دا هر څۀ د پېسو دپاره کوي- او دوي دروغ وائي- هغه جاوېد هوميوپيتهي ډاکټر له بوځي- خو جاوېد د هغوي نه هم نااُميده شي- جاوېد چونکې يو تعليم يافته تجربه لرونکې شخصيت وي، نو ځکه هغه د خپلو خپلوانو او دوستانو په مشورو خو په مختلفو ناتجربه کارو ډاکټرانو او حکيمانو باندې ګرځي – خو هغه د دوي په مشورو هېڅ کله هم عمل نۀ کوي او نۀ ئې دوايانوې استعمالوي -دې سره دهغۀ حالت نور هم خراب شي-يوه ورځ هغه د بې هوشۍ په حالت کښې هسپتال کښې هم داخل کړے شي-په هسپتال کښې چې هغۀ د تکليف او کړاؤ نه ډکې کومې ورځې شپې تېرې کړې نو هغې په وجه دجاوېد په زړۀ ډېر غټ اثرپرېوتو-

admitted to the hospital in a state of unconsciousness - in the hospital where he spent days and nights full of pain and suffering. It had a great .impact on Javed's heart

Sadness

Now Javed's heart and mind had this thing with certainty - that he is a diabetes patient - and this disease will not end forever - he also knew that he is a separate person from other people. This - and this disease is his companion for the rest of his life - he spent a few days in great sadness - but finally he started thinking about living with diabetes - he improved his diet - his I hope that many other people like me have diabetes . Reference has been .started

In short, Javed was patient with his condition - it had been six months of his illness - but even now his sugar level would increase - but his heart did not accept that sugar cane is the only treatment even in - this period

look up

خفګان

اوس د جاوېد زړۀ او ذهن ته دا خبره په یقین سره پرېوتې وه- چې هغه د شوګر مریض دے- او دا بیماري د همېشه دپاره چرې هم نۀ ختمېږي- هغۀ ته دا هم پته وه چې هغه د نورو انسانانو نه یو اړخ ته جدا انسان دے- او هم دا مرض د هغۀ د ټول ژوند ملګرے دے- څو ورځې خو ئې ښې خفګان کښې تېرې کړلې- خو آخر ئې د شوګر د مرض سره په ژوند تېرولو سوچ شروع کړو- هغۀ په خپل خوراک سخاک کښې بهتري راوسته- د هغۀ امیدؤ چې زما په شان نورو ډېرو خلکو ته هم د شوګر بیماري ده- هغۀ په خپل خوراک کښې هم تر ډېره حده ښۀ بدلون راوستو- په خوراک کښې خو به ئې خوړو ته ګوتې هم نه وروړلې- په دفترکښې ئې هم د کار د بدلون په حواله غور شروع کړے ؤ-

لنډه دا چې جاوېد اوس په خپل حال صبر کولو- د هغۀ د بیمارۍ شپږ میا شتې شوې وې- ولې اوس به هم د هغۀ شوګر مقدار زیاتېدو-خو زړۀ ئې نۀ منله چې ګني په دې دور کښې هم د شوګر بس هم دغه علاج دے-

لټون

جاوېد د یو پوهه او تکړه ډاکټر په لټون کښې ؤ- په دې هلو ځلو کښې څۀ موده تېره شوه دې دوران کښې جاوېد د

Javed was looking for a knowledgeable and talented doctor - some time passed in this effort, during this time Javed met with many doctors - these doctors included government, private and even military doctors - such a The doctor did not treat Javed, but there was no .improvement

Finally, Javed came to know how different treatment centers are treating diabetes - he saw everything with his own eyes, and of course, everything was not satisfactory in .any way

It may be that he had lost his courage a long time ago, but a secret strength was giving him courage. It was also known that there are many qualified and moral doctors in this institution serving the patients - still Javed was surprised at one point - if these stories are true, then does Javed need to go there? -----Days passed by

One day, Javed went to Karachi for some work of the company - on the way back, when he sat in the plane, his heart sank - and his whole body

ډېرو ډاکټرانو سره ولیدل- په دغه ډاکټرانو کښې سرکاري، پرائېویټ او تر دې چې فوځي ډاکټران هم شامل ؤ- داسې یو ډاکټر نۀ ؤ پاتې چانه چې جاوېد علاج نۀ وي کړے- خو بیائې هم هېڅ ښېګړه ونۀ شوه-

آخر جاوېد په دې خبره ور سېدۀ چې د علاج مختلف مرکزونه څۀ رنګ د شوګر د مرض علاج کوي- دا هر څۀ هغۀ په خپلو سترګو ولېدل او یقیناً چې دا هر څۀ په یو لحاظ هم اطمېنان بخش نۀ ؤ-

کېدے شي چې هغۀ ډېر پخوا حوصله بائېللے وے خو یو پټ طاقت هغۀ له حوصله ورکوله- یوه ورځ جاوېد د یوې معالجې اداري باره کښې ډېرې ښې خبرې واورېدې- کومه چې د خوش قسمتۍ نه هم په دغه ښار کښې جوړه کړې شوې وه- هغه په دې هم خبر ؤ چې په دغه اداره کښې ډېر قابلان او با اخلاقه ډاکټران د مریضانو خدمت کښې لګیا دي- بیا هم جاوېد په یو دوه کښې حېران ؤ- چې کۀ چرې دا قیصې رښتیا وي نو آیا جاوېد له هلته تلل پکار دي؟ ورځې تېرېدې-----

یوه ورځ جاوېد د کمپنۍ د څۀ کار په سلسله کښې کراچۍ ته لاړو- واپسې کښې چې کله په جهاز کښې کښېناستۀ نو د هغۀ زړۀ په ډوبېدو شو- او ټول بدن ئې خولې خولې شو- د جاوېد په خوا کښې ناست یو کس ائېرهوسټس ته د چینو راوړلو دپاره

began to sweat - a person sitting next to Javed said to the air hostess. He said to bring China, because Javed's sugar was low" - Erhostus brought China and Javed gave him a spoon (spoon) of China, then Javed's nature was restored - Javed, the person whose name was Khalid Mahmood, told him how It turns out that my sugar is - and this time my sugar was low -

Khalid said that I am a model diabetic and have the ability to .control my sugar

A light of hope

When Javed heard Khalid's words, he was very impressed and happy that he got a light of hope - because of Khalid, new ways of treatment were opened for Javed - he also advised Javed to create a model diabetic. - And he also attached the institution where knowledgeable, skilled and honest doctors like Dr. Khurram are always ready to serve the patients of .diabetes

On this, Javed was very satisfied and reassured - that there is someone who knows the address of my problem - and - understands my problem

ووئېل، ځکه چې د جاوېد شوګر کم شوے ؤ"- ائېرهوسټس چینې راوړل او جاوېد تهئې یو کاشوغه (چمچ) چینې ورکړل نو د جاوېد طبیعت بحال شو- جاوېد هغه کس چې خالد محمود نوم ئې ؤ ورته ووئېل چې تاسو ته څنګه معلومه شوه چې زما شوګر دے- او دا وخت زما شوګر کم شوے ؤ-

خالد ووئېل چې زۀ ماډل ډایابیټک یمماته په خپل شوګر د قابو ساتلو مهارت حاصل دے-

د اُمید رڼا

جاوېد چې د خالد خبرې واورېدې نو هغه ډېر متاثره شو او خوشحاله هم شو چې هغۀ ته د امید یوه رڼا مېلاؤ شوه- د خالد په وجه جاوېد ته د علاج دپاره نوې نوې لارې پرانستې شوې- هغۀ جاوېد ته د ماډل ډایابیټک جوړېدو مشوره هم ورکړه- او هغه ادارې ئې ورته هم په نښخه کړې چرته چې د ډاکټر خرم غوندې پوهه، ماهر او ایماندار ډاکټران د شوګر د مریضانو خدمت ته هروخت چمتو وي-

په دې جاوېد ته ډېر اطمېنان او ډاډګېرنه مېلاؤ شوه- چې څوک خو شته چاته چې زما د مسئلې پته ده- او زما په مسئله پوهیږي-

خالد هم د شوګر مریض وي اوجاوېد ته ئې ووئېل چې زۀ هم د یو سسټم سره منسلک یم- په دې سسټم کښې مریض چرې هم ځانګړتیا نه محسوسوي- بلکې د ډاکټرانو پوره یو

Khaled is also a diabetes patient and told Ojaved that I am also connected to a system - in this system, the patient does not feel special anywhere - but a whole team of doctors is involved in this procedure - the patients themselves -They help each other<br>Javed will be very happy and he will also become a part of this system and start his treatment from the Diabetes Institute. And take his face"<br>- Awaiting print	ټیم په دغه طریقه کار عمل کولو کښې شامل وي- مریضان خپله د یو بل سره امداد کوي- جاوېد ډېر زیات خوشحاله شي او دغه شان هغه هم ددې سستم یوه برخه شي- او دډایابیټکس انسټیټیوټ نه خپل علاج شروع کړي- جاوېد د یو ماډل ډایابیټکس جوړېدو کوشش څنګه کوي------- دې دپاره ددې کتاب "د شوګر رنځ او د هغهٔ مخ نیوے" د چاپ انتظار کوئ-
