# Supplementary material for: An innovative state-of-the-art health storytelling technique for better management of type 2 diabetes
Source: Front Public Health. 2023 Sep 29;11:1215166. doi: 10.3389/fpubh.2023.1215166 (PMC10585594; doi:10.3389/fpubh.2023.1215166)
Supplement: Supplementary file 2 [file Data_Sheet_2.PDF]

Supplementary File S2

Story Recall Questions

1. Do you remember the name of the storybook? Describe<br>2. Do you remember 12 names of the characters in the storybook? Describe<br>3. Do you remember the main events in the story? Describe<br>4. What are the features of the main character X in the story?<br>5. What was the story about?<br>6. Who advised the main character X of the story to join the Diabetes Club?<br>7. For how long did the main character X of the story remain unaware that he had been suffering from Diabetes?<br>8. Which company the main character X was working for?<br>9. How many children the main character X had?<br>10. Do you remember who was the person whom the main character X met in Diabetes club for the first time?<br>11. How many doctors the main character X did meet?<br>12. What is the full name of the main character X?<br>13. Who was the person whom the main character X met in the market place?<br>14. What special medicine was recommended by the “Hakeem” for diabetes control?<br>15. What was the reaction of the main character X to the advice of one of his friends regarding diabetes?<br>16. What was the name of the doctor who gave the main character X the advice to have some leave from the office and take some rest?<br>17. What was the sugar level of the main character X when checked by a technician at a clinic?<br>18. Name the favorite dish of the main character X, which he found difficult to skip?<br>19. What was the name of the office colleague of the main character X, who was not taking his disease seriously and said it was just an excuse not to come to office?<br>20. How many family members the main character X had?	1. کیا آپ کو کہانی کی کتاب کا نام یاد ہے؟ بیان کریں۔<br>2. کیا آپ کو کہانی کی کتاب کے 12 کرداروں کے نام یاد ہیں؟ بیان کریں۔<br>3. کیا آپ کو کہانی کے اہم واقعات یاد ہیں؟ بیان کریں۔<br>4. کہانی میں مرکزی کردار X کی خصوصیات کیا ہیں؟<br>5. کہانی کیا تھی؟<br>6. کہانی کے مرکزی کردار X کو ذیابیطس کلب میں شامل ہونے کا مشورہ کس نے دیا؟<br>7. کہانی کا مرکزی کردار X کتنی دیر تک اس بات سے بے خبر رہا کہ وہ شوگر کے مرض میں مبتلا ہے؟<br>8. مرکزی کردار X نے کس کمپنی کے لیے کام کیا؟<br>9. مرکزی کردار X کے کتنے بچے تھے؟<br>10. کیا آپ کو یاد ہے کہ وہ شخص کون تھا جس سے مرکزی کردار X پہلی بار ذیابیطس کلب میں ملا تھا؟<br>11. مرکزی کردار X نے کتنے ڈاکٹروں سے ملاقات کی؟<br>12. مرکزی کردار X کا پورا نام کیا ہے؟<br>13. وہ شخص کون تھا جس سے مرکزی کردار X بازار میں ملا تھا؟<br>14. ذیابیطس کے کنٹرول کے لیے "حکیم" نے کون سی خاص دوا تجویز کی؟<br>15. شوگر کے حوالے سے اپنے ایک دوست کے مشورے پر مرکزی کردار X کا ردعمل کیا تھا؟<br>16. اس ڈاکٹر کا کیا نام تھا جس نے مرکزی کردار X کو دفتر سے چھٹی لینے اور آرام کرنے کا مشورہ دیا تھا؟<br>17. کلینک میں ٹیکنیشن کے ذریعے چیک کرنے پر مرکزی کردار X کا شوگر لیول کیا تھا؟<br>18. مرکزی کردار X کی پسندیدہ ڈش کا نام بتائیں، جسے چھوڑنا اسے مشکل لگا؟<br>19. مرکزی کردار X کے دفتری ساتھی کا کیا نام تھا، جو اپنی بیماری کو سنجیدگی سے نہیں لے رہا تھا اور کہتا تھا کہ یہ دفتر نہ آنے کا صرف ایک بہانہ تھا؟<br>20. مرکزی کردار X کے خاندان کے کتنے افراد تھے؟
