# Supplementary material for: An innovative state-of-the-art health storytelling technique for better management of type 2 diabetes
Source: Front Public Health. 2023 Sep 29;11:1215166. doi: 10.3389/fpubh.2023.1215166 (PMC10585594; doi:10.3389/fpubh.2023.1215166)
Supplement: Supplementary file 3 [file Data_Sheet_3.PDF]

Supplementary File S2

Development and validation of a tool to assess the Literary Parameters of a Health Story that Makes a health Story Interesting and Catchy to the Patients Readers – The First Unique Literary Devices Assessment Scoring' (LDAS-Tool)

Summary

Problem considered: Health story development is an emerging technique that seeks behavioral changes in the patients to achieve health targets. Health stories are developed by professional writers to engage patients to adhere to health advice, guidelines, prescriptions and recommendations. Health story writing for health communication purposes is not that much simple because a health story is much different from a common fiction story. There are many challenges one may face while developing a story for health communication. A big challenge usually faced by a health story writer or developer while compiling the story maybe staying true to the story vs. being evidence-based. Beside strictly adhering to the modem scientific facts presented in a story, it is also essential to take care of literary elements while compiling a health story. Without literary elements, there would be no literature and without no literature a health story is likely to be not catchy enough to attract the readers and make them believe in the contents presented in the story. While understanding literary elements are essential for a health story and while there are numerous tools available to evaluate the scientific evidence presented in the story, no tool is available that can assess the literary parameters of a health story. Methods: Therefore, we aimed to develop and validate a tool that can assess the most 15 important literary parameters of a health story (Allusion, Diction, Alliteration, Allegory, Colloquialism, Euphemism, Flashbacks, Foreshadowing, Imagery, Juxtaposition, Metaphor/simile, Personification, Onomatopoeia, Symbolism, and Tone). Following a comprehensive literature search, an initial twenty-one-item scale was developed and was subjected for content validation to a panel of experts (n=10). Results: The final scale was reduced to 15 items as six items were removed and/or merged together. The item level content validity index (I-CVI) and scale level content validity index (S-CVI) was found to be 80%. The Cronbach's alpha score was 0.8, suggesting a satisfactory co-efficient of reliability. Conclusion: The scale to assess the overall literary quality of the health story was developed and validated.

Literary Devices Assessment Scoring' (LDAS-Tool)

Short Description	Full Description	YES	NO
Allusion	There is substantial material that connect the reader to the story		
Diction	There is substantial material that give choice of words or style used by the writer in order to convey the message		
Alliteration	There is substantial material that uses the same letters or sounds at the beginning of words in a sentence or title		
Allegory	There is substantial material that shows that abstract ideas are described using characters, events, or other elements.		
Colloquialism	There is substantial material that shows expressions, words, and phrases that are used in informal, everyday speech, including slang.		
Euphemism	There is substantial material that shows actually any terms that refer to something impolite or unpleasant		
Flashbacks	There is substantial material that shows the narrator goes back in time for a specific scene or chapter in order to give more context for the story		
Foreshadowing	There is substantial material that shows the author places elements within the writing that gives clues about what will happen in the future of the story		
Imagery	There is substantial material that shows use visually descriptive or figurative language in your writing		
Juxtaposition	There is substantial material that shows "human-like qualities to non-human elements"		
Metaphor/simile	There is substantial material that show placing contrasting elements next to one another in order to emphasize one or both, including words, scenes, or themes		
Personification	There is substantial material that shows comparisons used to create better clarification and understanding for readers		
Onomatopoeia	There is substantial material that shows that the word or phrase that shows you the *sound* something makes		
Symbolism	T There is substantial material that shows there is substantial material that shows use of a situation or element to represent a larger message, idea, or concept		
Tone	There is substantial material that shows conveys the narrator's opinion, attitude, or feelings about what is written		

Note: Please Tick 'Yes' or 'No'

References

Rahmonova, U. (2022). ANALYSIS OF POETIC DEVICES. *CURRENT RESEARCH JOURNAL OF PHILOLOGICAL SCIENCES*, *3*(07), 20-24.

Sirwah, K. S. (2022). Dramatic Allusion and Referencing Literary Tradition in Jez Butterworth's The Ferryman. *CDELT Occasional Papers in the Development of English Education*, *79*(1), 45-67.

Aduko, P. A., & Kogri, E. N. Exploring the Characterizationand the Literary Devices Used in the Narration of the GurenɛFolktale.

Foulani, H., & Abidi, A. (2022). *Allusions within TS Eliot's The Wasteland* (Doctoral dissertation, University Ahmed DRAIA of Adrar).

Supplementary File S2

Development and validation of a tool to assess the Scientific Quality of a Health Story for Diabetic Patients– The First Unique Scientific Quality Assessment Scoring (SQAS-Tool)

Problem considered: Health story development is an emerging technique that seeks behavioral changes in the patients to achieve health targets. Health stories are developed by professional writers to engage patients to adhere to health advice, guidelines, prescriptions and recommendations. Health story writing for health communication purposes is not that much simple because a health story is much different from a common fiction story. There are many challenges one may face while developing a story for health communication. A big challenge usually faced by a health story writer or developer while compiling the story maybe staying true to literary parameters of the story vs. being evidence-based. While writing a health story, it is very much possible that the literary parameters that are equally needed while compiling a story may overshadow the scientific facts presented in the story. While we appreciate that without no literary elements, there would be no literature and without no literature a health story is likely to be not catchy enough to attract the readers and make them believe in the contents presented in the story. However, it is also essential to present the scientific facts in a story with its true spirit – a fact which necessitate the existence of a tool that may be used to evaluate the integrity and accuracy of the scientific facts presented in the story. No tool is available that can assess the accuracy parameters of a health story. Methods: Therefore, we aimed to develop and validate a tool that can assess the most 17 important literary parameters of a health story (*Authoritative/Informed, Known Sources, Within Context Sources, Objectivity of the Sources, Source Attribution, WHO Guidelines, Nutrition Advice, Physical Activity Advice, Medication Advice Life-style Advice, No Conflict of Interest, Cost Effective, Benefits Quantified, Side-effects Declared Alternativity, Availability, and Novelty).* Following a comprehensive literature search, an initial 20 items scale was developed and was subjected for content validation to a panel of experts (n=12). Results: The final scale was reduced to 17 items as 3 items were removed and/or merged together. The item level content validity index (I-CVI) and scale level content validity index (S-CVI) was found to be 80%. The Cronbach's alpha score was 0.8, suggesting a satisfactory co-efficient of reliability. Conclusion: The scale to assess the scientific quality of the health story was developed and validated.

Scientific Quality Assessment Scoring (SQAS-Tool)

Short Description	Full Description	YES	NO
Authoritative/Informed	the source either has the credentials or is close to the event/story and can provide accurate information (the innermost circle).		
Known Sources	the source and their affiliations are explicitly provided in the story.		
Within Context Sources	The source's evidence is presented in context.		
Objectivity of the Sources	The source's quotes, evidence, and observations are presented accurately and objectively.		
Source Attribution	The source is given proper attribution in the story.		
WHO Guidelines	The information provided about diabetes are according to the criteria set by the WHO		
Nutrition Advice	The advice provided on nutrition is scientific and sound		
Physical Activity Advice	The advice provided on Physical activity is scientific and sound		
Medication Advice	The advice provided on medication is scientific and sound		
Life-style Advice	The advice provided on overall lifestyle is scientific and sound		
No Conflict of Interest	The story is free of any conflict of interest		
Cost Effective	The story shows the cost of the intervention is feasible and comparable		
Benefits Quantified	The benefits of intervention are explicitly quantified		
Side-effects Declared	Any side effects/inverse effects are declared		
Alternativity	The intervention compares the new approach with existing alternatives		
Availability	The intervention is readily available		
Novelty	The intervention establishes the true novelty of the approach		

Note: Please Tick 'Yes' or 'No'

References

Vickers, P. (2022). *Identifying Future-Proof Science*. Oxford University Press.

Moleman, M., Jerak-Zuiderent, S., van de Bovenkamp, H., Bal, R., & Zuiderent-Jerak, T. (2022). Evidence-basing for quality improvement; bringing clinical practice guidelines closer to their promise of improving care practices. *Journal of Evaluation in Clinical Practice*, *28*(6), 1003-1026.

Sandoval, W. A., & Millwood, K. A. (2005). The quality of students' use of evidence in written scientific explanations. *Cognition and instruction*, *23*(1), 23-55.

Mueller, S. M., Hongler, V. N., Jungo, P., Cajacob, L., Schwegler, S., Steveling, E. H., ... & Brandt, O. (2020). Fiction, falsehoods, and few facts: cross-sectional study on the content-related quality of atopic eczema-related videos on YouTube. *Journal of medical Internet research*, *22*(4), e15599.

Supplementary File S2

CRAAP (assessment of currency, relevance, authority, accuracy, and purpose)

Score	0	1	2	3
**Currency**	No indication of date/time of th events presented in the story	time of most of the events look <5 years old	time of most of the events look <2 years old	time of most of the events look <1 years old
**Relevance**	It does not address pertinent aspects of diabetes	It provides some information, but is not enough, or is not the right type of information.	It provides most of the pertinent information but is not comprehensive.	Provides all pertinent information.
**Authority**	The author is not an expert and has no relevant credentials. Or the organization is not known.	Author has ties to the topic but no relevant credentials. Or the organization is of questionable authority.	Author has credentials related to the topic but not expertise is field is not high. Or, the organization is well-known, but the degree of expertise is not high.	The author's credentials are given and clearly indicate that he/she is an expert. Or the organization is well-known and highly credible.
**Accuracy**	Information is provided with no indication as to where it comes from.	Poor quality sources are cited, or information is entirely from personal experience.	Some information is obtained from quality sources.	All information is obtained from clearly stated, high quality sources.
**Purpose**	The purpose is to present a biased point of view, sell or promote an idea, service, or product. It is not factual or a balanced point of view. The opinion is either not backed up with facts of the facts are distorted.	The purpose of is to sell or promote something, but it also provides some quality information. Or expresses opinion that is somewhat logical with some evidence.	The purpose is to educate or offer mostly factual information. Expressed opinion is mostly logical and evidence based.	The purpose is to provide information of a scholarly, academic, or high-quality nature. Evidence for opinion is factual, presented as numbers in charts, graphs, tables, or statistics, or with adequate evidence for the opinion.

References

Lewis, A. B. (2018). Tips from the Experts. *Issues in Science and Technology Librarianship*.

Berg, C. (2017). Teaching website evaluation: The CRAAP test and the evolution of an approach.

Supplementary File S4

Development and validation of a tool to assess the Scientific Quality of a Health Story for Diabetic Patients– The First Unique Scientific Quality Assessment Scoring (SQAS-Tool)

Problem considered: Health story development is an emerging technique that seeks behavioral changes in the patients to achieve health targets. Health stories are developed by professional writers to engage patients to adhere to health advice, guidelines, prescriptions and recommendations. Health story writing for health communication purposes is not that much simple because a health story is much different from a common fiction story. There are many challenges one may face while developing a story for health communication. A big challenge usually faced by a health story-writer or developer while compiling the story may be staying specific to the disease the story is written about. A story targeting diabetic patients should give diabetic-specific information in order to make the diabetic patients believe in the content of the story as more relevant to diabetes. Otherwise, if a wide range of topic related to other issues not closely relevant to diabetes presented in the story may likely detract the attention of diabetic patients and hence the story may lose its impact. It is, therefore, essential to evaluate a health story written exclusively for diabetes related health knowledge communication that whether it presents information that are specific to diabetes or not. No tool is available that can assess diabetes-specific information of a health story. Methods: Therefore, we aimed to develop and validate a tool that can assess the most 20 important diabetic-specific questions related to disease include (Specific Causes, specific signs/symptoms, specific tests, Single Sign Specificity, Time Specificity, specific nutritional management/treatment, Specific dose of CHO, specific strict diabetic diet, specific protein diet, Specificity of CHO diet, Specific multi-vitamins/minerals, specific nutritional complications, Specific Ketoacidosis Symptoms, Specificity on ketone elimination, Specificity on insulin/dosage timing, Specific diet plan, Diabetes specific diet-related other complications, Specific Physical Activity/Exercise, Specific diet-drug interaction, specific self-care recommendations*)*. Following a comprehensive literature search, an initial 20 items scale was developed and was subjected for content validation to a panel of experts (n=12). Results: The final scale was reduced to 17 items as 3 items were removed and/or merged together. The item level content validity index (I-CVI) and scale level content validity index (S-CVI) was found to be 80%. The Cronbach's alpha score was 0.8, suggesting a satisfactory co-efficient of reliability. Conclusion: The scale to assess the scientific quality of the health story was developed and validated.

Diabetes Specific Question Scoring (DSQS-Tool)

	Questions		Yes	No
Specific Causes	What are the common but specific nutritional causes of DMT2?	This question is addressed in the story		
specific signs/symptoms	What are the specific signs/symptoms of nutritional deficiencies of DMT2	This question is addressed in the story		
specific tests	How is DMT2 evaluated and/or diagnosed using specific tests?	This question is addressed in the story		
Single Sign Specificity	Should I be concerned about being diabetic with the following readings: Fasting blood sugar between 80 to 100; 200 one hour after a meal?	This question is addressed in the story		
Time Specificity	When should medical and nutritional care be sought for DMT2?	This question is addressed in the story		
specific nutritional management/treatment	What is the specific nutritional management/treatment of DMT2?	This question is addressed in the story		
Specific dose of CHO	How many carbohydrates per meal or a day would be good for a diabetic?	This question is addressed in the story		
specific strict diabetic diet	Does a specific strict diabetic diet mean eliminating sugar totally from the diet?	This question is addressed in the story		
specific protein diet	Is a diet high in specific protein diet best for a diabetic?	This question is addressed in the story		
Specificity of CHO diet	Why should the daily carbohydrate intake be consistent for DMT2?	This question is addressed in the story		
Specific multi-vitamins/minerals	What brand of multi-vitamin/minerals is best for someone with diabetes?	This question is addressed in the story		
specific nutritional complications	What are the specific nutritional complications of DMT2?	This question is addressed in the story		
Specific Ketoacidosis Symptoms	What is ketoacidosis and what are its symptoms?	This question is addressed in the story		
Specificity on ketone elimination	How do I make sure to get rid of ketones in my system after an illness?	This question is addressed in the story		
Specificity on insulin/dosage timing	When does a diabetic patients need Insulin?	This question is addressed in the story		
Specific diet plan	What is the best dietary plan for a diabetic patients?	This question is addressed in the story		

Note: Please Tick 'Yes' or 'No'

References

Vaughan, L. (2005). Dietary guidelines for the management of diabetes. *Nursing Standard (through 2013)*, *19*(44), 56.

Raj, G. D., Hashemi, Z., Contreras, D. C. S., Babwik, S., Maxwell, D., Bell, R. C., & Chan, C. B. (2018). Adherence to diabetes dietary guidelines assessed using a validated questionnaire predicts glucose control in adults with type 2 diabetes. *Canadian Journal of Diabetes*, *42*(1), 78-87.

Burant, C. (Ed.). (2012). *Medical management of type 2 diabetes*. American Diabetes Association.
